# Gender-based stereotyping and cost discrepancies for razors

**DOI:** 10.1016/j.ijwd.2021.01.022

**Published:** 2021-02-05

**Authors:** Michelle J. Chang, Shari R. Lipner

**Affiliations:** a Drexel University College of Medicine, Philadelphia, PA, United States; b Weill Cornell Medicine, Department of Dermatology, New York, NY, United States

**Keywords:** Razor, Cost discrepancy, Price disparity, Patient education, Gender stereotypes, Skin of color

Dear Editors:

Dermatologists suggest hair removal methods for pseudofolliculitis barbae, folliculitis, and hirsutism (Somani and Turvy, 2014). Gender-related cost discrepancies are well documented in personal care products (New York City Department of Consumer Affairs, 2015). Our objectives were to investigate gender-based price differences and marketing for razors.

The three largest e-commerce retailers selling disposable razors (Amazon, Walmart, Target) were reviewed between June 30, 2020 and July 16, 2020. Brand, price, blade number, gender specification (the word “men” or “women”, or image on packaging), colors, lubrication strip, and handle/head features were recorded. When a razor’s price differed between retailers, the mean price was used. Price per razor, grouped by blade number, was compared between men and women. Marketing images were categorized by Fitzpatrick skin type. Two-tailed *t* tests and χ^2^ tests were performed.

We identified 176 razors: 83 for men, 86 for women, and 7 gender neutral. Women’s four-blade razors were priced 66% higher than men’s ($3.02/razor vs. $1.94/razor; *p* = .005). Women’s five-blade razors were priced 47% higher than men’s ($5.14/razor vs. $4.03/razor; *p* = .047; Table 1). No significant differences were found in the number and cost of men’s and women’s razors with lubrication strips, pivoting heads, or special features (dermatologist tested, hypoallergenic, sensitive skin).

**Table 1:** features and average prices of women’s and men’s razors

Blades, n	Prices averaged, n		Average price per razor, $		*p*-value	Scented razors, n		Razors with a trimmer/edging blade, n	
	Women	Men	Women	Men		Women	Men	Women	Men
1	2	1	1.00	0.99	N/A				
2	9	12	1.27	0.84	.26				1
3	26	29	2.29	1.92	.45	7	1		2
4	18	13	3.02	1.94	.005	6		1	3
5	30	25	5.14	4.03	.047	3		2	17
6	1	3	3.00	2.86	N/A				4

N/A, not applicable.

Of the razors for men and women, 76 (92%) and 82 (95%) stated “men” or “women”, respectively, in the title/description. Sixty-three women’s products contained marketing images: 50 (79%) were of skin types I/II, 7 (11%) of III/IV, and 10 (15%) of V/VI. Of the 57 men’s razors with images, 49 (86%) depicted skin types I/II, 5 (9%) III/IV, and 9 (16%) V/VI. Some marketing included multiple models.

Our study demonstrated that, on average, women’s four- and five-blade razors were more expensive than men’s. Women’s razors were more likely to be scented than men’s (χ^2^ [1; n = 169] = 15.3; *p* < .001), which may have affected production costs. However, cost differentials are likely negligible because men’s razors are more likely to feature trimmers/edging blades (χ^2^ [1; n = 169] = 25.8; *p* < .001). Men may replace razors more often; a 2019 survey showed that more men (6190 of 17,536 men [35%]) than women (1139 of 19,484 women [6%]) shaved once or more daily (Statistica, 2020). Gender-related price differences were identified in other personal care products. Women paid 40% more for identical topical minoxidil products, and women’s facial moisturizers were priced $3.09 higher than men’s (Manatis-Lornell et al., 2019, Wehner et al., 2017).

Razor colors adhered to traditional gender stereotypes and disproportionately represented white and binary populations. Men’s razors more often had bold and darker colors and women’s pastel/lighter colors (Fig. 1). This stereotyping parallels facial moisturizer marketing, with men’s packaging having darker colors and hard edges and women’s featuring lighter colors (Manatis-Lornell et al., 2019). Ninety-three percent of razor marketing listed “women” or “men” and excluded nonbinary individuals. Packaging rarely depicted darker skin colors.

**Fig. 1:**
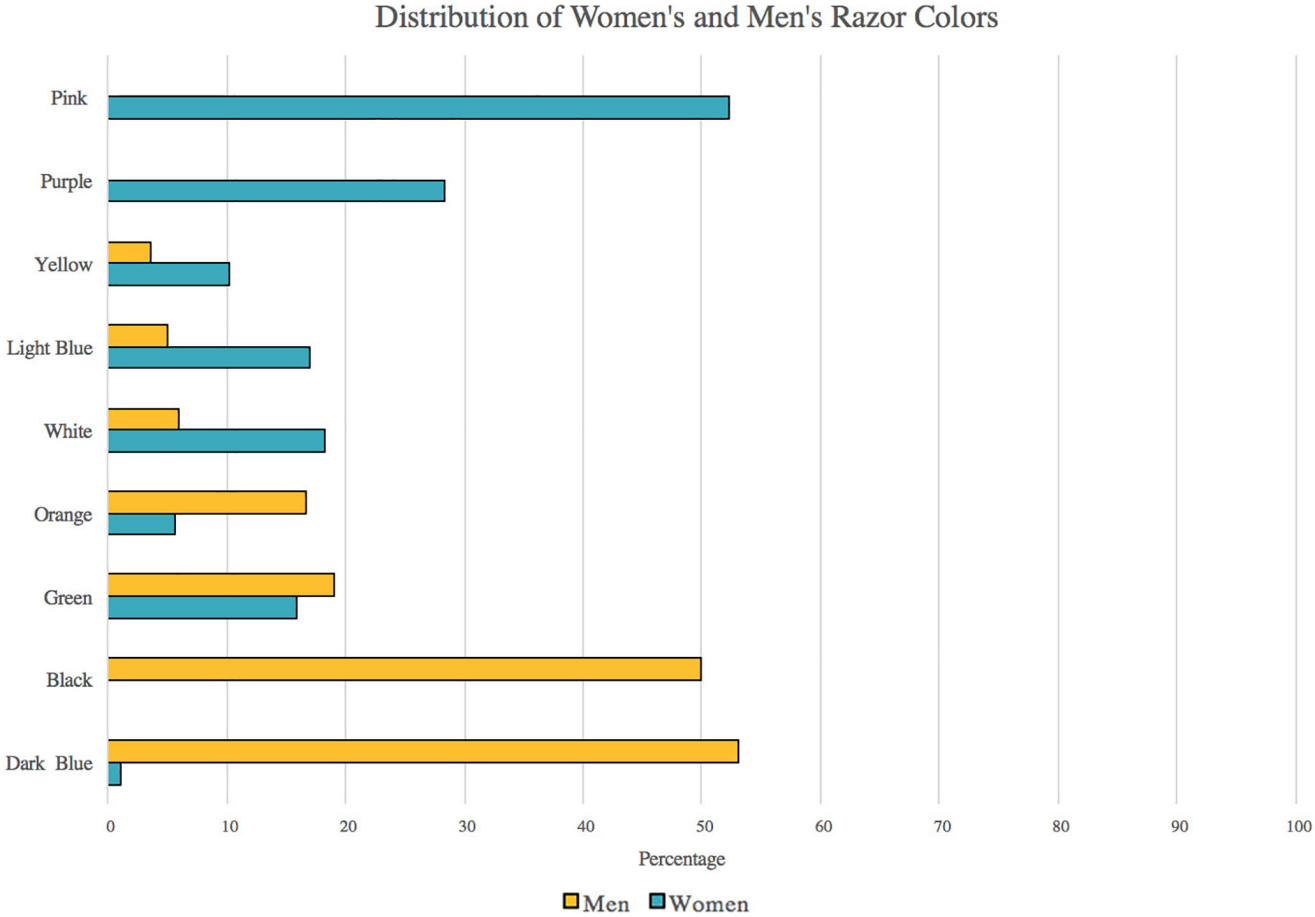
Distribution of women’s and men’s razor colors. Men’s razors were mostly black (42 of 83; 48%), dark blue (44 of 83; 53%), green 16 of 83; 18%), and orange (14 of 83; 16%). Women’s razors were pink (46 of 86; 52%), purple (25 of 86; 28%), white (16 of 86; 18%), light blue (15 of 86; 17%), and green (14 of 86; 16%). Percentages do not add to 100% because some razors had multiple colors.

This study has several limitations. The sample sizes grouped by blade number were small. Size, design, packaging, and blade/handle replacements were not analyzed. Also, assigning Fitzpatrick skin types is subjective.

Our study demonstrated gender-based price disparities and stereotyping and underrepresentation of darker skin types for razors. We advocate for physician and consumer awareness of inequitable razor pricing and marketing.

## Conflicts of interest

None.

## Funding

None.

## Study approval

The author(s) confirm that any aspect of the work covered in this manuscript that has involved human patients has been conducted with the ethical approval of all relevant bodies.
